# Terahertz refractive phenotype of living cells

**DOI:** 10.3389/fbioe.2022.1105249

**Published:** 2023-01-10

**Authors:** Guangxu Zhang, Yadi Wang, Jiang Qian, Yue Wang, Xueling Li, Junhong Lü

**Affiliations:** ^1^ Shanghai Institute of Applied Physics, Chinese Academy of Sciences, Shanghai, China; ^2^ Jinan Microecological Biomedicine Shandong Laboratory, Jinan, China; ^3^ University of Chinese Academy of Sciences, Beijing, China; ^4^ School of Pharmacy, Binzhou Medical University, Yantai, China; ^5^ Shanghai University of Medicine and Health Sciences, Shanghai, China; ^6^ Shanghai Advanced Research Institute, Chinese Academy of Sciences, Shanghai, China

**Keywords:** terahertz spectroscopy, refractive index, water droplet, living cells, droplet microfluidic

## Abstract

Cellular refractive index is a vital phenotypic parameter for understanding the cell functional activities. So far, there remains technical challenges to obtain refractive index of viable cells at the terahertz frequency in which contains rich information closely related to their physiological status. Here we introduce a label-free optical platform for interrogating cellular phenotypes to measure the refractive index of living cells in near-physiological environments by using terahertz spectroscopy with the combination of cellular encapsulation in a confined solution droplet. The key technical feature with cells encapsulated in aqueous droplets allows for keeping cellular viability while eliminating the strong adsorption of solvent water to terahertz signal. The obtained high signal-to-noise ratio enables to differentiate different cell types (e.g., *E. coli*, stem cell and cancer cell) and their states under stress conditions. The integrating of terahertz spectroscopy to droplet microfluidic further realizes automated and high-through sample preparation and detection, providing a practical toolkit for potential application in cellular health evaluation and phenotypic drug discovery.

## Introduction

Cell refractive index, an important phenotypic parameter that correlates with the biological properties such as internal mass (Gul et al., 2021), has been widely used as marker in the field of cell biology and biomedicine to determine the cell types and investigate cellular activities (Zhuo et al., 2011; Liu et al., 2016). With the rapid development of advanced optical techniques, the measurement of refractive index has been extended to a wider frequency range (Zhuo et al., 2011; Liu et al., 2016). Compared with the visible region, the refractive index in the terahertz (THz) region is non-linear (Baxter and Guglietta, 2011; Tcypkin et al., 2019; Novelli et al., 2020) and reveals the reorientation dynamics of water that hydrates the biomolecules (Tros et al., 2017; Peng et al., 2021), which contains rich information closely related to the cellular activity and physiological status (Yang et al., 2016a; Ball, 2017; Liu et al., 2019; Zhang et al., 2021; Lou et al., 2022). Also intriguing is that THz wave does not cause ionizing damage making its more safe in biological measurements (Olga et al., 2021; Liao et al., 2022). Therefore, a pressing need is to develop a rapid and widely applicable way to measure the THz refractive index of living cell with improved the signal-to-noise ratio.

In principle, the refractive index at THz frequencies of substance can be measured by THz time-domain spectroscopy (THz-TDS) (Baxter and Guglietta, 2011). Several pioneering works have demonstrated the possibility to achieve cellular refractive index by using THz spectroscopy. For example, Yang et al. (2016b) reported that refractive index varied between different species of bacterial colonies. Wang et al. (2019) found that various cells in nervous system had different refractive indices, which would increase after cancerization. Cao et al. (2021) proposed that refractive index could be used to distinguish different cancer cells. However, due to the aqueous medium required for living cells measurement easily produces strong background signal interference (Peng et al., 2020), there remains a big technical challenge for obtaining the refractive index of cells in a viable state.

**FIGURE 1:**
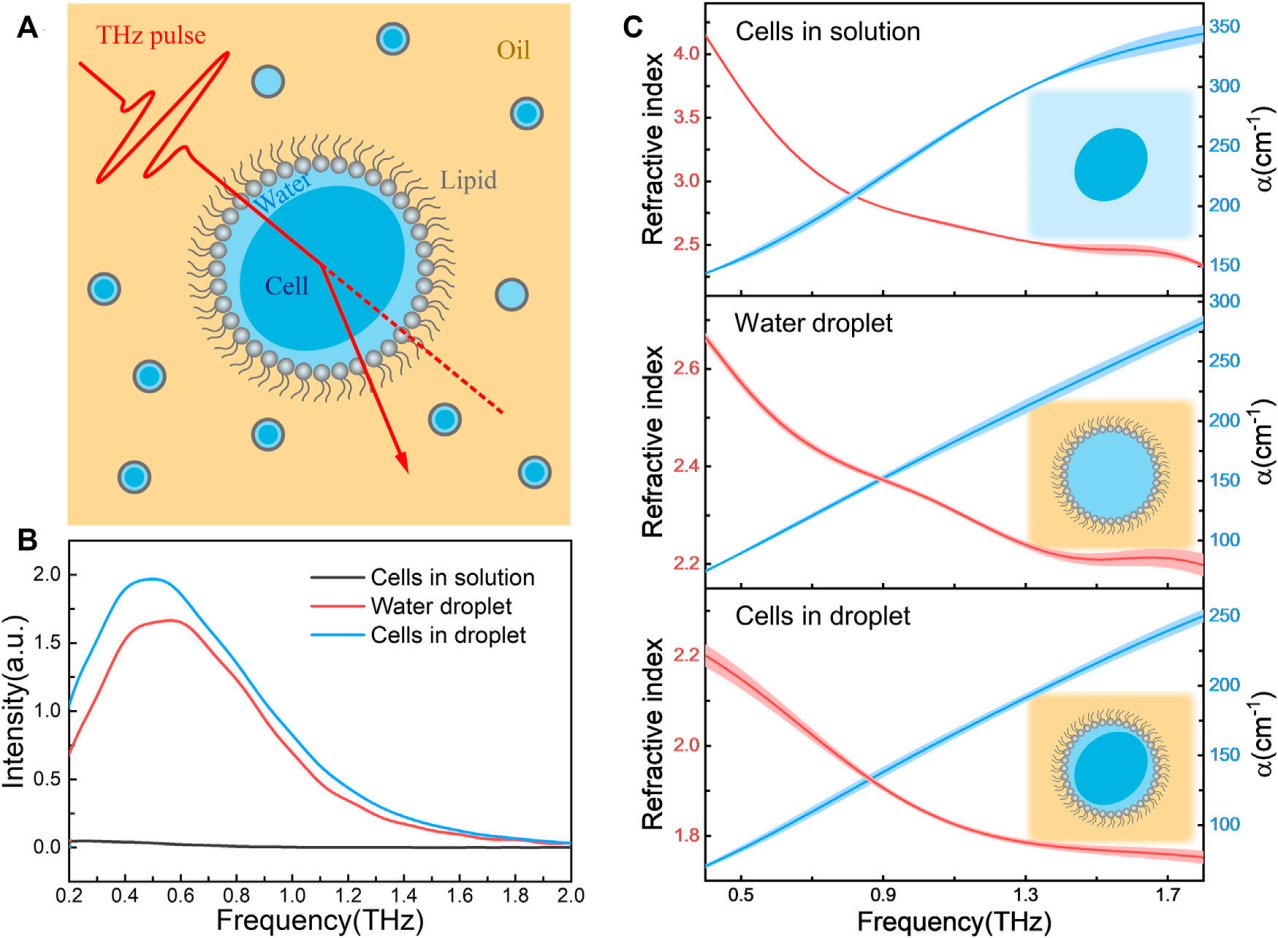
**(A)** Schematic of refractive index measurement platform based on the combination of terahertz spectroscopy and cellular droplet sampling. The cell-containing droplets (water phase) were dispersed in the reagent transparent to THz signals (oil phase), and the phospholipids at the oil-water interface served as surfactants; **(B)** The transmission spectra of *E. coli* in aqueous solution (cells in solution), droplet containing TBS solution alone (water droplet) and *E. coli*-containing droplet (cells in droplet). All *E. coli* samples had the same bacterial concentration of 9.0 × 10^10^ cfu/mL; **(C)** The refractive index (left axis) and absorption coefficient (α, right axis) spectra of these three samples, where the schematic diagram of the forms of samples are shown in insert. The shaded area indicating the error bars with triplicated measurements.

Herein, we introduce a technical platform for determining the refractive index of cells in an aqueous condition by using the combination of THz-TDS and droplet sampling (Figure 1A). Specifically, the cell samples were encapsulated one by one in aqueous droplets constructed by the self-assemble phospholipids (Yang et al., 2019; Tang et al., 2021; Tang et al., 2022). The cellular encapsulation provides not only a liquid environment for keeping cellular viability (Sart et al., 2022) but also allows for eliminating the strong adsorption of solvent water to THz signal (Weisenstein et al., 2021). Due to the droplets, consisting of the cells and a little confined water around, are surrounded by reagents with high transmittance in THz region, the signal attenuation of the sample mainly attributes to the adsorption of intracellular water. Thus, from the THz signal, we can easily calculate the absorption coefficient (α) and the refractive index of cell samples. Using the optical platform, we have successfully obtained the refractive index of three kinds of cell types, including *E. coli*, stem cell and cancer cell, and their states under stress conditions. We also demonstrated the integrating of this method into a droplet microfluidics chip to acquire an automated and high-through preparation and detection toolkit for the application practicability.

## Results and discussion

### Refractive index measurement of living cells encapsulated in aqueous droplets

Figure 1B shows the obtained THz signal of samples in three forms, *E. coli* in buffer solution (cells in solution), droplet containing buffer alone (water droplet) and droplet containing *E. coli* (cells in droplet). Due to the strong absorption of solvent water, the transmission THz electric field strength of cells in bulk solution is very weak, almost consistent with the transmission spectrum of buffer itself (Supplementary Figure S3A). In contrast, for samples in droplet forms the signal-to-noise ratio was improved more than an order of magnitude, and the significant spectral difference was observed between samples with and without cells. The refractive index and absorption coefficient spectra of these three samples were shown in Figure 1C. We found that the optical properties of the cells in solution were nearly same as the buffer alone (Supplementary Figure S3B), suggesting the THz signal are mainly derived from the bulk aqueous solution. However, when the buffer droplets was confined in lipid monolayer, a cell-like environment (Shang and Zhao, 2021), the refractive index significantly decreased comparing to that bulk solution. Interestingly, when the cells were encapsulated in the droplet solutions, the refractive index was further lower down. Since the THz signal of cell-containing droplets might mainly attribute to the intracellular water (Folpini et al., 2017) and little confined water outside the cells, the obtained refractive index is reasonable to describe the physical parameter of living cells themselves.

### Terahertz refractive index for cell functional activity assay

**FIGURE 2:**
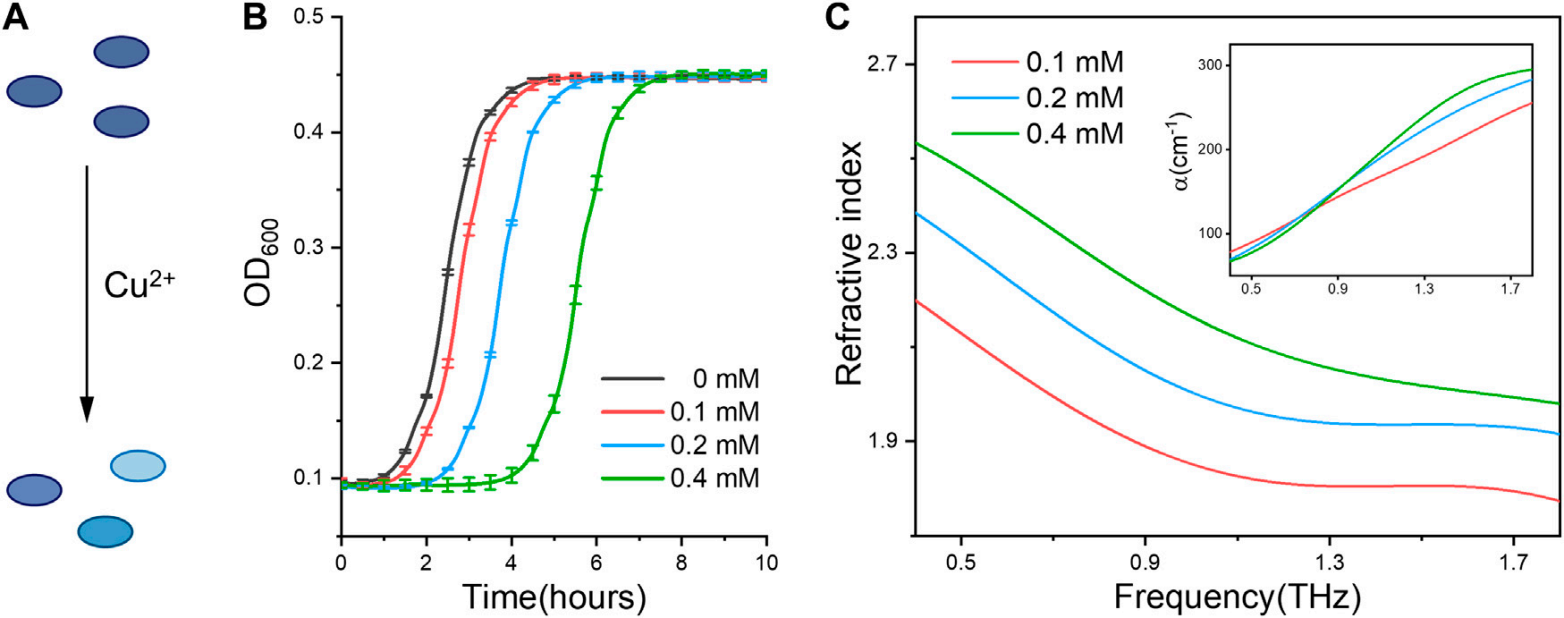
**(A)** Schematic showing Cu^2+^ ions treatment induces distinct activity states of bacterial cells. **(B)** The growth curve of *E. coli* under 0, .1, .2 and .4 mM of Cu^2+^ treatment, respectively. **(C)** Refractive index and absorption coefficient spectra (inset) of *E. coli* cells. All bacterial samples were treated with Cu^2+^ and accounted to the same cell numbers for THz measurement.

Using the established methodology, we ask if the measured refractive index can be used to discriminate the cellular states under different physiological conditions. For this purpose, we firstly compared the bacterial (*E. coli*) cells subjected to metal ions stress. Since trace copper ions are required for keeping the bacterial viability (Giachino and Waldron, 2020), *E. coli* cells under different states were obtained with Cu^2+^ treatment at sublethal concentrations (Figure 2A). From the growth curves (Figure 2B), we can find the bacteria treated under higher Cu^2+^ concentration exhibit longer delayed growth time (lower viability), indicating cellular viable states in an ion concentration dependent manner. Interestingly, bacterial cells with different viable states have distinct physical parameters, including the refractive index and absorption coefficient spectra shown in Figure 2C. Especially, the cellular refractive index increases with the higher concentration of ions treatment. In other words, the cells with lower viability, either induced by different ion concentrations or treatment times (Supplementary Figure S4), have a bigger refractive index. For example, at the .9 THz frequency, the cellular refractive indexes are 1.89, 2.05 and 2.22 under Cu^2+^ treatments with 0.1 mM, 0.2 mM, 0.4 mM, respectively.

**FIGURE 3:**
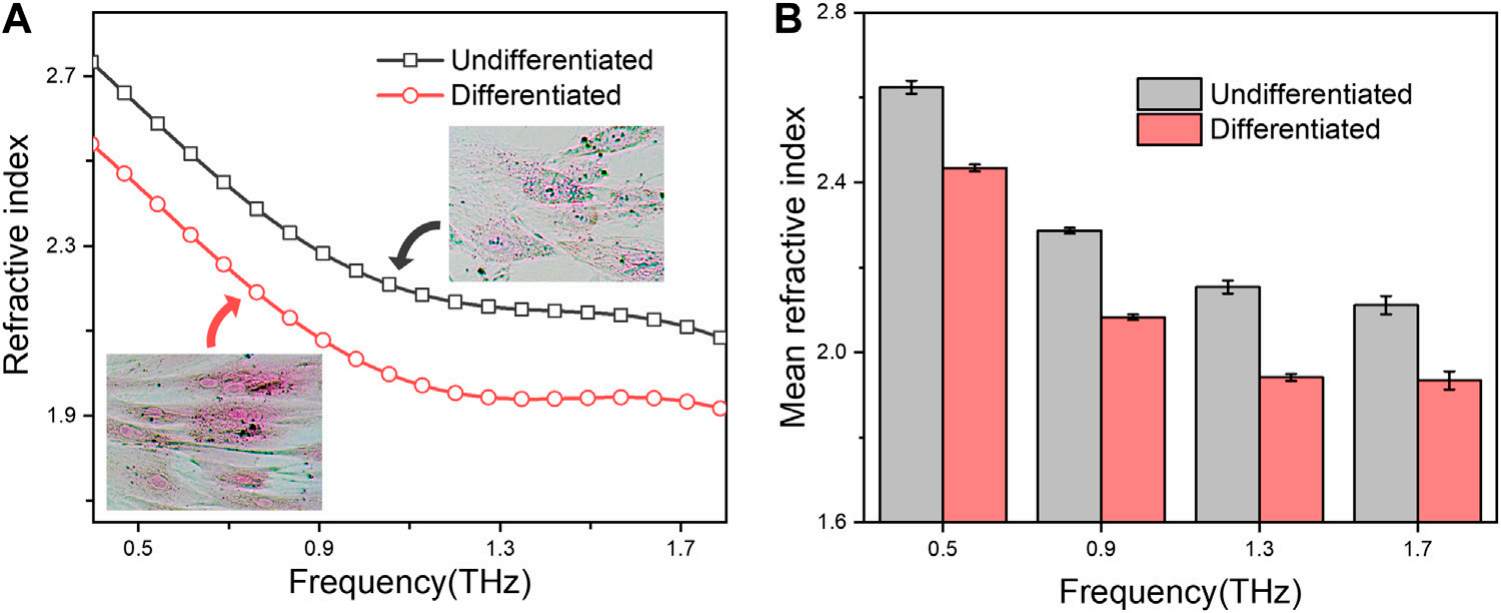
**(A)** Refractive index spectra of undifferentiated or osteogenic differentiated MSCs in droplets. The microscopy photograph of the corresponding cells after alizarin red S staining are shown in insert; **(B)** Mean refractive index at .5, .9, 1.3 and 1.7 THz. All groups were set to the same cell concentration of 1.0 × 10^5^ cells/well.

We further test whether the functional stages of eukaryotic cells would be also reflected through these terahertz parameter measurements. To this end, two states of mesenchymal stem cells (MSCs) during osteogenic differentiation were choose for the investigation. The osteogenic differentiation of MSCs led to successively form mineralization of bone matrix (Khezri et al., 2021), showing an intense alizarin red staining (Figure 3A inset; Supplementary Figure S5), in which hydration plays a vital structuring role across the bone hierarchy (Surowiec et al., 2022). As shown in Figure 3, the refractive index of osteogenic differentiated MSCs is smaller than that of undifferentiated ones at the measured terahertz frequency (from .4–1.8 THz). Thus, these results not only indicated that the refractive index changes during the differentiation process of stem cell, but also proved the developed technique is suitable for kinds of cell types.

Taking together, these two experiments clearly demonstrated the feasibility and repeatability of our method to represent the cellular states, either for bacterial cells or eukaryotic cells, by quantitative measurement of their terahertz refractive indexes, providing a novel label-free technique for evaluating the cellular physiological or pathological activities.

### Integrating of microfluidics chip for droplet production and optical detection

**FIGURE 4:**
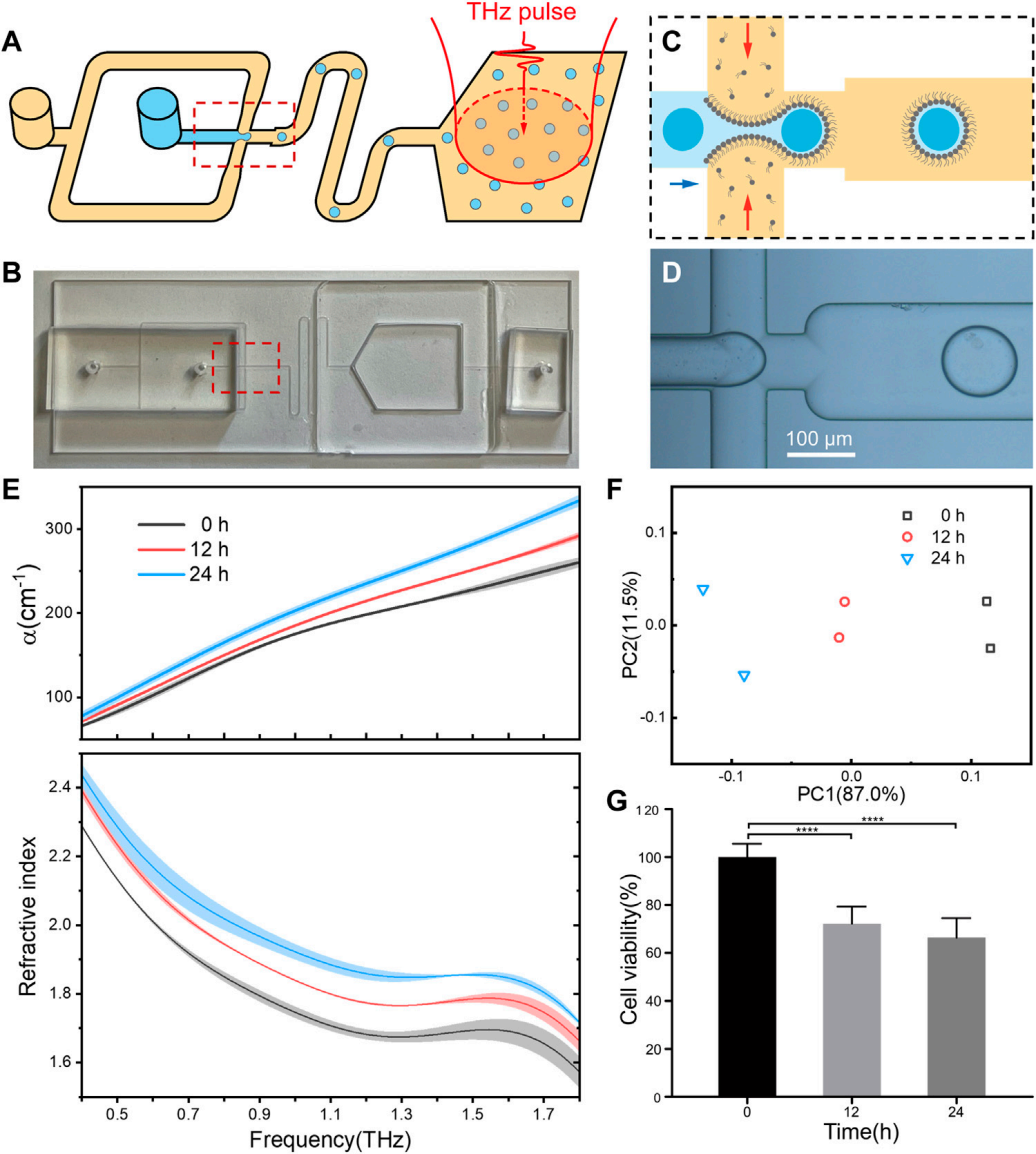
The integrating of droplet microfluidics for automated and high-through performance. **(A)** Schematic of the microfluidic device that can generate droplet sample and perform THz-TDS detection. An aqueous phase containing cells was passed through a flow-focusing junction where it met an oil phase containing lipids, leading to the generation of droplets. Red arrows show the position of a cell before and after encapsulation. Droplets were collected in a quartz chamber which severed as the terahertz measurement cuvette. **(B)** The optical image of the microfluidic chip. **(C,D)** The schematic and optical image of the droplet generation process. **(E)** Refractive index spectra of the droplets containing HepG2 cells after treatment by resveratrol for 0, 12 and 24 h. All groups were set to the same cell concentration of 1.4 × 10^5^ cells/well. For each group of cells, the droplets were prepared and measured twice, and the shaded area indicates the error bars. **(F)** Principal component analysis (PCA) plots of the measured refractive index. **(G)** Cell viability of resveratrol treated HepG2 cells.

Next, we try to integrate droplet formation and terahertz detection into a microfluidics chip for practical applications. In our experiment, a two-module microfluidics device were constructed to generate cell-containing droplets and optical measurements, synchronously (Figures 4A, B). In the cell encapsulation module, an aqueous phase containing living cells was passed through a flow-focusing junction where it met an oil phase containing lipids, leading to the generation of cell-contained droplets (Figures 4C, D). The flow rate and junction geometry were carefully chosen to control the droplet radius for full encapsulation of individual cells inside (Ding et al., 2020). After passing through a flow channel in a sufficient time to get stabilization, the droplets were collected in the detection module, which was fabricated by quartz windows with well-transmission of THz wave. Once enough cells sample were measured, their optical parameters could be achieved.

In a proof-of-concept study, the microfluids chip was applied to analyze the relationship between terahertz refractive index and drug response of living cells. As expected, following with a low-concentration resveratrol treatment, the measured cellular refractive index was increasing accordingly to treatment time (Figure 4E), while the cell viability decreased as indicated in MTT assay (Figure 4G). Principal component analysis (PCA) clearly indicated that the cellular refractive index is high related to the effect of drug treatment (Figure 4F). Although more kinds of drugs and cell lines are still required to setup a universal rule on the relationship of drug effect with cellular refractive index, the integration of terahertz spectroscopy and microfluidic chip paves a brand-new way for drug screening based on cellular refractive phenotypes.

## Conclusion

In summary, we have developed a novel strategy and method to measure the refractive index of living cells in near-physiological environment by using terahertz spectroscopy with the combination of cellular encapsulation in a confined solution droplet. The advantage of cell confinement lies on its ability to improving signal-to-noise ratio meanwhile keeping cellular viability. Using this technical platform, we have successfully obtained the refractive index of *E. coli*., mesenchymal stem cell, and liver cancer cell. Importantly, the high sensitivity and good repeatability of this approach enables to discriminate the cellular states and stress responses, such as the stem cells under differentiation conditions, the cancer cells with drug treatment, et al. Furthermore, the technique is easily integrated into a microfluidics chip in which the droplet sampling and optical measurement are simultaneously achieved, demonstrating the key step towards automation and high-through application. This novel technology development not only offers a valuable toolkit to understanding the fundamental role of the intracellular water in cell biology but also provides a label-free optical approach for bioanalytical applications in cellular health evaluation and phenotypic drug discovery.

## Materials and methods

### Microfluidic device fabrication

The soda-lime glass microfluidic chip was fabricated by conventional photolithography, wet etching and thermal bonding (Baker and Roper, 2010). A glass with chrome layer and AZ-1505 (from AZ technology) were used as a photoresist to create a master. The glass was then etched with a 66:14:20 (v:v:v) mixture of H_2_O:HNO_3_:HF. After that, access holes of 1 mm diameter were machined using a diamond-tipped drill bit. The chip was cleaned, hydrolyzed and put together under running water for sealing. The chip was then thermal bonding at 500°C.

The two-module microfluidic chip, designed in AutoCAD. The oil phase reagent entered through a wide (200 μm) inlet channel. Cells entered through an inlet channel with a width of 90 μm, then entered a cross-shaped flow-focusing junction (90 μm × 90 μm) where the droplets generated. The droplets then flowed through a meander with 180 μm width and 100 mm total length to sufficiently stabilize the lipid interface. The above microstructures constituted the first module, where the depth of channel was 40 μm. The droplet sample then entered the detection module, a chamber with a height of 1 mm and an area of 1 cm^2^, equipped with a waste outlet. In order to ensure the transparency of the cavity in the THz range, laser cutting was used to remove the glass substrates above and below the cavity and quartz plates were glued as substitute substrates. The thickness of the quartz plate is .5 mm, and the distance between them is the same as the height of the cavity (1 mm). To assist the entry/exit of liquid, PDMS pedestals are provided at the inlet and outlet.

### Preparation of droplet samples

Reagents for cell culture, including Tris-buffered saline (TBS), culture medium, etc., were purchased from Sinopharm Chemical Reagent (China) unless otherwise specified. DOPC (1,2-dioleoyl-sn-glycero-3-phosphocholine, purchased from Avanti Polar lipids, Inc.) and anhydrous hexadecane (purchased from Sigma-Aldrich) were used as surfactant and oil phase reagent, respectively. Cell suspensions at the indicated concentrations were used as the aqueous phase. For drug-treated cells, they were washed after treatment and transferred to drug-free TBS or culture medium. See Supplementary Material for more details of cell culture procedures.

Chloroform solution of DOPC evaporated under nitrogen gas and dried in vacuum to obtain the thin film of lipids, which was then dissolved in hexadecane to a concentration. The aqueous droplets were fabricated according to the following procedures: the prepared cell suspensions were injected in the phospholipid/hexadecane solutions (45 mg/mL), and the aqueous droplet samples would be obtained by vortex, where the dispersed phase accounted for a volume fraction of 9% for bacteria-containing droplets and 10% for droplets contains other cells. When preparing droplet samples containing eukaryotic cells, vigorous mechanical manipulation should be avoided to prevent cell rupture. When microfluidic chip was used to prepare droplet samples, the oil phase was DOPC dissolved in hexadecane (4 mg/mL) and the water phase was the cell suspension with the pump flow rate at 8 and 1 μL/min, respectively. See Supplementary Material for more details of microfluidic device operation.

### THz spectroscopy

The samples were measured using a THz time-domain spectroscopy (THz-TDS) system. See Supplementary Material for the details of the system. In the experiments, samples were placed in a fused quartz cuvette with a thickness of .5 mm (or the quartz detection window of the microfluidic chip with the same thickness), and the sample chamber was controlled at a temperature of 21.0°C ± .5°C and humidity within 1%. Transmission mode was applied for all measurements. The empty cuvette was used first as a reference signal. The absorption coefficient, $\alpha \left(\nu \right)$ , and the refractive index, $n\left(\nu \right)$ , were then obtained as follows:

$$\alpha \left(\nu \right)=d^{-1}In\left[I_{r}\left(\nu \right)/I_{s}\left(\nu \right)\right]$$

$$n\left(\nu \right)=n_{r}\left(\nu \right)+\frac{c}{2\pi \nu d}\left[\phi_{s}\left(\nu \right)-\phi_{r}\left(\nu \right)\right]$$

Where d is the optical path length of the cuvette, *I*_*r*_(*ν*) and *I*_*s*_(*ν*) are the intensity of reference and sample solutions, *n*_*r*_(*ν*) is the refractive index of the reference, *Φ*_*s*_(*ν*) and *Φ*_*s*_(*ν*) are the phase of reference and sample, respectively. The refractive index of the water phase was further obtained by a binary component model, see Supplementary Material for the details. PCA was conducted using R language without any preprocessing of the measured refractive index.

## Data Availability

The original contributions presented in the study are included in the article/Supplementary Material, further inquiries can be directed to the corresponding author.
