# Supplementary material for: Terahertz refractive phenotype of living cells
Source: Front Bioeng Biotechnol. 2023 Jan 10;10:1105249. doi: 10.3389/fbioe.2022.1105249 (PMC9871359; doi:10.3389/fbioe.2022.1105249)
Supplement: Supplementary file 1 [file Table1.DOCX]

**Supplementary Information**

**Terahertz refractive phenotype of living cells**

Guangxu Zhang^a,b,f^, Yadi Wang^b,c^, Jiang Qian^a,b,f^, Yue Wang^a,b,f^, Xueling Li^b,d^, Junhong Lü^a,b,c,d,e*^

^a^Shanghai Institute of Applied Physics, Chinese Academy of Sciences, Shanghai 201800, China

^b^Jinan Microecological Biomedicine Shandong Laboratory, Jinan 250000, China

^c^School of Pharmacy, Binzhou Medical University, Yantai 264003, China

^d^Collaborative Research Center, Shanghai University of Medicine and Health Sciences, Shanghai 201318, China

^e^Interdisciplinary Research Center, Shanghai Advanced Research Institute, Chinese Academy of Sciences, Shanghai 201203, China

^f^University of Chinese Academy of Sciences, Beijing 100049, China

Correspondence information:

Junhong Lü, Collaborative Research Center, Shanghai University of Medicine and Health Sciences, Shanghai 201318, China, [lvjunhong@jnl.ac.cn](mailto:lvjunhong@jnl.ac.cn)

**Table of contents:**

**Part 1.** Terahertz time-domain spectroscopy (THz-TDS) measurement.

**Part 2.** Cell culture and microfluidic device operation.

**Fig.S1.** Optical path diagram of the THz-TDS used in the experiment.

**Fig S2.** The transmission spectra of empty cuvette, buffer droplet and buffer solution.

**Fig.S3.** The refractive index and absorption spectra of hexadecane.

**Fig S4.** A comparison of the original spectrum and the fitted spectrum of (a) absorption coefficient and (b) refractive index.

**Fig.S5.** Terahertz spectra of aqueous buffer solution and *E. coli* in aqueous buffer solution.

**Fig.S6.** The growth curve and Refractive index spectra of *E. coli* treated with 0.4 mM Cu^2+^ for 5, 10 and 20 min.

**Fig.S7.** The microscopy photograph of the undifferentiated and osteogenic differentiated MSCs after alizarin red S staining.

**Part 1. Terahertz time-domain spectroscopy (THz-TDS) measurement**

The THz-TDS system used in the experiment was equipped with a Mai Tai femtosecond laser with a wavelength of 800 nm, a pulse width of less than 100 fs, a repetition frequency of 80 MHz, and an average power of 700 mW as the light source. The optical path diagram of the system was shown in Fig.S1. The emitted femtosecond laser is divided into two paths by a dichroic prism for THz wave excitation and coherent detection, respectively. By controlling the movement of the electric translation platform through software programming, the optical path difference between the excitation light and the detection light can be precisely controlled, so that the detection light can be manipulated to scan and sample the terahertz pulse and obtain the complete terahertz time domain spectra. The system is based on photoconductive antennas to generate and receive terahertz signals. The electric field of the THz beam as a function of time E_THz_(t) was recorded, and the frequency dependent power and phase of the transmitted pulse was produced after a fast Fourier transformation.

The cell-containing droplets were formed by dispersing low volume fraction (9%) of *E. coli* suspensions in hexadecane. Fig. S2 (a) and (b) shows the refractive index (RI) and absorption coefficient of water and hexadecane, respectively. Briefly, hexadecane exhibits very different optical properties from water, especially having a very low terahertz absorption coefficient. Fig. S3 and Fig. S4 show the obtained THz signals of the *E. coli* samples in suspension and droplet, respectively. Although the experimental system utilized a relatively high-power THz source (700 mW), the THz electric field strength of samples in suspension is very weak (Fig. S3(a)). This is apparently due to the excess absorption of water. Simultaneously, no significant change can be observed in the transmission spectra with different bacterial concentrations (Fig. S4(a)), further indicating poor quantitation performance. In contrast, for droplet samples, the THz electric field exhibits a much greater strength (Fig. S3(b)), with an increase of more than an order of magnitude in the frequency-domain signal (Fig, S4(b)). In particular, the transmission spectra show an obvious concentration dependent, suggestion a much-improved SNR. Apart from the less water fraction of droplet samples, the signal enhancement also owing to confinement of the aqueous droplet on its restricted water. The significant improvement of SNR makes it possible to obtain the refractive index of living cells.

For droplet samples, the measured refractive index is a combination value of the oil and water phase, and the refractive of the latter can be calculated using effective medium model. One of the widely employed effective medium theories in the THz region is the linear refractive index model based on the Lambert Beer law, which stipulates the effective refractive index of the mixed sample as the weighted average of each constituent’s refractive index weighted by its volume fractions^1, 2^, resulting in the equation:

$$n_{eff}=\frac{n_{hex}V_{hex}+n_{aq}V_{aq}}{V_{hex}+V_{aq}}$$

$$\alpha_{eff}=\frac{\alpha_{hex}V_{hex}+\alpha_{aq}V_{aq}}{V_{hex}+V_{aq}}$$

Where n_eff_ and α_eff_ are the measured refractive index and absorption coefficient, n_hex_/α_hex_ and n_aq_/α_aq_ are the refractive index/ absorption coefficient of hexadecane and the water phase (i.e., the droplet), respectively, and V_hex_ and V_aq_ are the volume fraction of hexadecane and the water phase in the mixed sample. It should be noted that all effective medium models may have small deviations from the exact value^3^, but this does not prevent the comparison of the relative magnitudes of the refractive indices of different samples.

**Part 2. Cell culture and microfluidic device operation**

**Cell culture and cell assay**

*Escherichia coli* (*E. coli*). The logarithmic *E. coli* CGMCC1.2463 was inoculated into Luria–Bertani broth (Sinopharm Chemical Reagent, Shanghai) and incubated at 37℃ with continuous shaking (240 rpm) until stationary growth phase. The preculture was washed three times with Tris-buffered saline (TBS) solution (Sinopharm Chemical Reagent, Shanghai) and the concentration was determined using the optical density at 600nm (OD600). The bacterial sample was subsequently enriched to the required concentration. For sample treated with Cu^2+^, the bacterial sample was mixed with CuCl_2_ solution with a final bacteria concentration of 3×10^9^ CFU/ml, and then incubated at 37℃ with continuous shaking (240 rpm) for a required time. The Cu^2+^-treated sample was also enriched to the required concentration after being washed with TBS solution. For the evolution of the growth curve, the washed bacterial was diluted to a concentration of 3×10^7^ CFU/ml, then inoculated into Luria–Bertani broth according to the volume ratio of 9:1 (200 μL). The OD600nm was recorded in the following 15 h at 37 °C with a microplate reader (VersaMax Microplate Reader), with 5 s auto shake before each measurement.

Primary human mesenchymal stem cells (hMSCs). The hMSCs (purchased from icell Biotechnology Co., Ltd) were grown in low glucose DMEM (Thermo Fisher) supplemented with 10% fetal bovine serum (GIBCO), 100 U/mL penicillin, and 100 μg/ mL streptomycin. Cells were incubated at 37 °C with 5% CO_2_ saturation in a humidified atmosphere. Culture medium was refreshed every 3 days, and cells were passaged to the sixth generation. To assess osteoblast differentiation, hMSCs (1000/well) were seeded onto 6 well plate and cultured in an osteoblast differentiation medium (low glucose DMEM with 10%FBS, 100nM dexamethasone, 10mM sodium β-Glycerophosphate, 50 μM ascorbic acid) the culture solution was replaced by fresh medium every 3 days. The MSCs were induced to osteoblast differentiation for 21 days. To prepare the cell suspension, a standard trypsinization protocol was performed with 2 mL of 0.25% trypsin-EDTA solution. The cell suspension was then centrifuged at 1000 rpm for 5 min and suspended in fresh culture medium. For staining experiments, the hMSCs were stained with 2% alizarin red S (Epizyme) at day 21. At first, the medium was removed and wells were washed with PBS twice. Then, the hMSCs were fixed with 4% paraformaldehyde for 15 min. After fixing, the wells were washed with distilled water twice. Next, the cells were stained with 2% alizarin red for 30 min. Subsequently, the cells were washed with distilled water twice. The stained samples were dried at room temperature.

HepG2 human liver cancer cells. The HepG2 cells (purchased from icell Biotechnology Co., Ltd) were cultured in DMEM containing 10% GIBCO and 5% streptomycin at 37℃ with 5% CO_2_ saturation in a humidified atmosphere. The DMEM was renewed every other day until the cells reached near-confluence (5-y days). A similar trypsinization protocol was conducted to prepare the cell suspension. To assess different cell viability, HepG2 cells (5000/well) were seeded onto 96 well plate and cultured in DMEM containing resveratrol (24 μg/ml) for different time. For MTT assay, 20 μl MTT solution was added to each well. After incubating for four hours, the liquid in the well was sucked out and 150 μl DMSO was added. After standing for ten minutes, the absorbance was read at 490 nm by microtiter plate reader. The absorbance was proportional to the cell viability, and the measured absorbance was subtracted from the absorbance of background wells and the survival rate of the cells treated for 0 hour was taken as 100%.

**Microfluidic device operation**

Before preparing the droplet samples, rain repellent (purchased from aquapel), a regent that makes glass completely hydrophobic, was injected into the channels of the microfluidic chip. After one minute incubation, the rain repellent was sucked out and the chip was washed several times with pure water and buffer. This step is to make the glass channels hydrophobic to form water-in-oil droplets. Polyethylene tubing was inserted in inlets and outlets at one end, and to 1 ml syringe on the other. The syringes were driven using syringe pumps (NE-4000, purchased from New Era Pump Systems Inc.). The oil phase was DOPC dissolved in hexadecane (4mg/ml), and the water phase was the cell suspension mixed well before operation. The pump flow rate was set at 8 and 1 μl/min for oil phase and water phase, respectively.

**
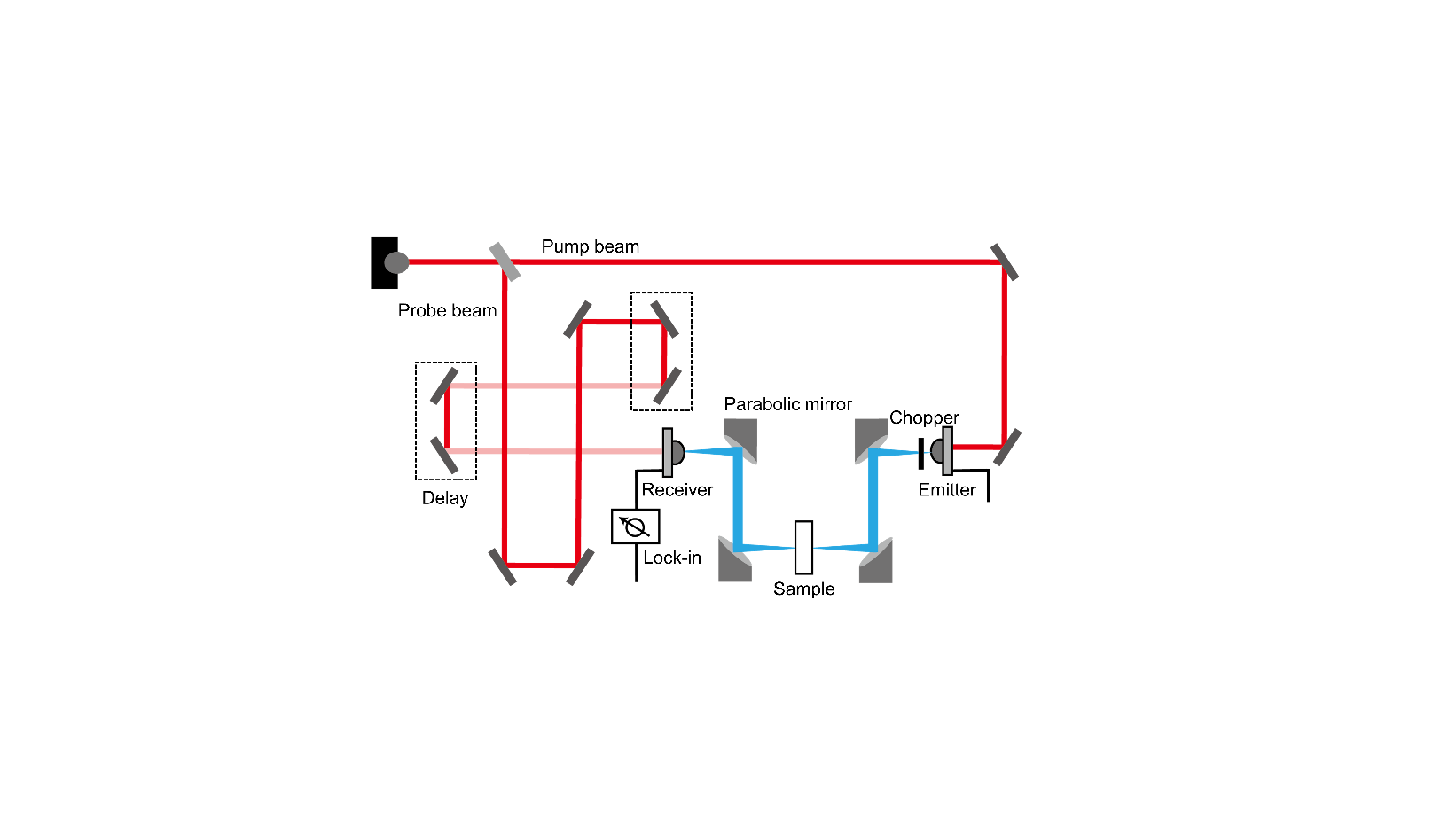
**

Fig S1. Optical path diagram of the THz-TDS used in the experiment.


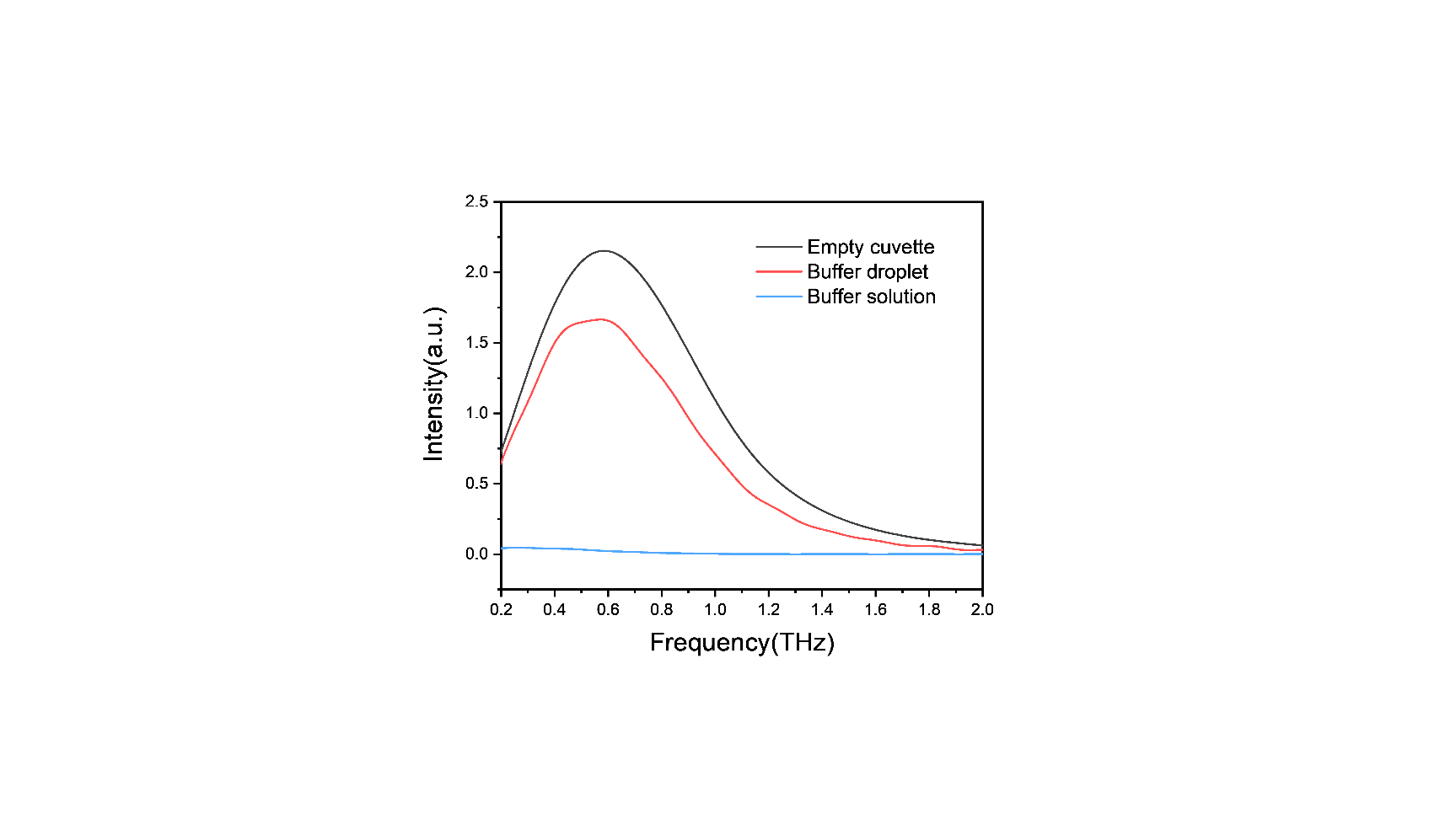


Fig S2. The transmission spectra of empty cuvette, buffer droplet and buffer solution.

**
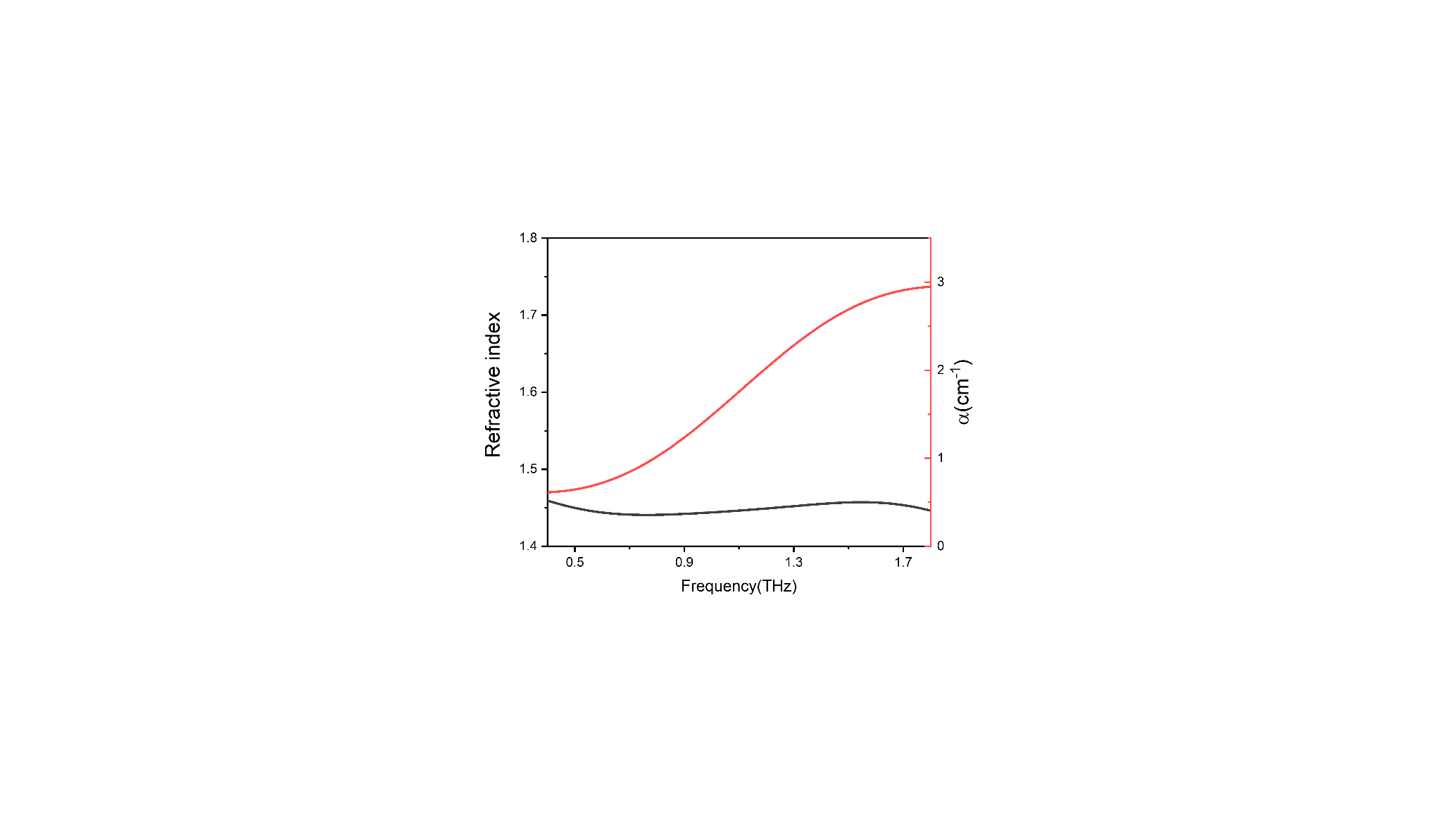
**

Fig S3. The refractive index and absorption spectra of hexadecane. The refractive index (black line) and absorption coefficient (red line) correspond to the left and right axes, respectively.


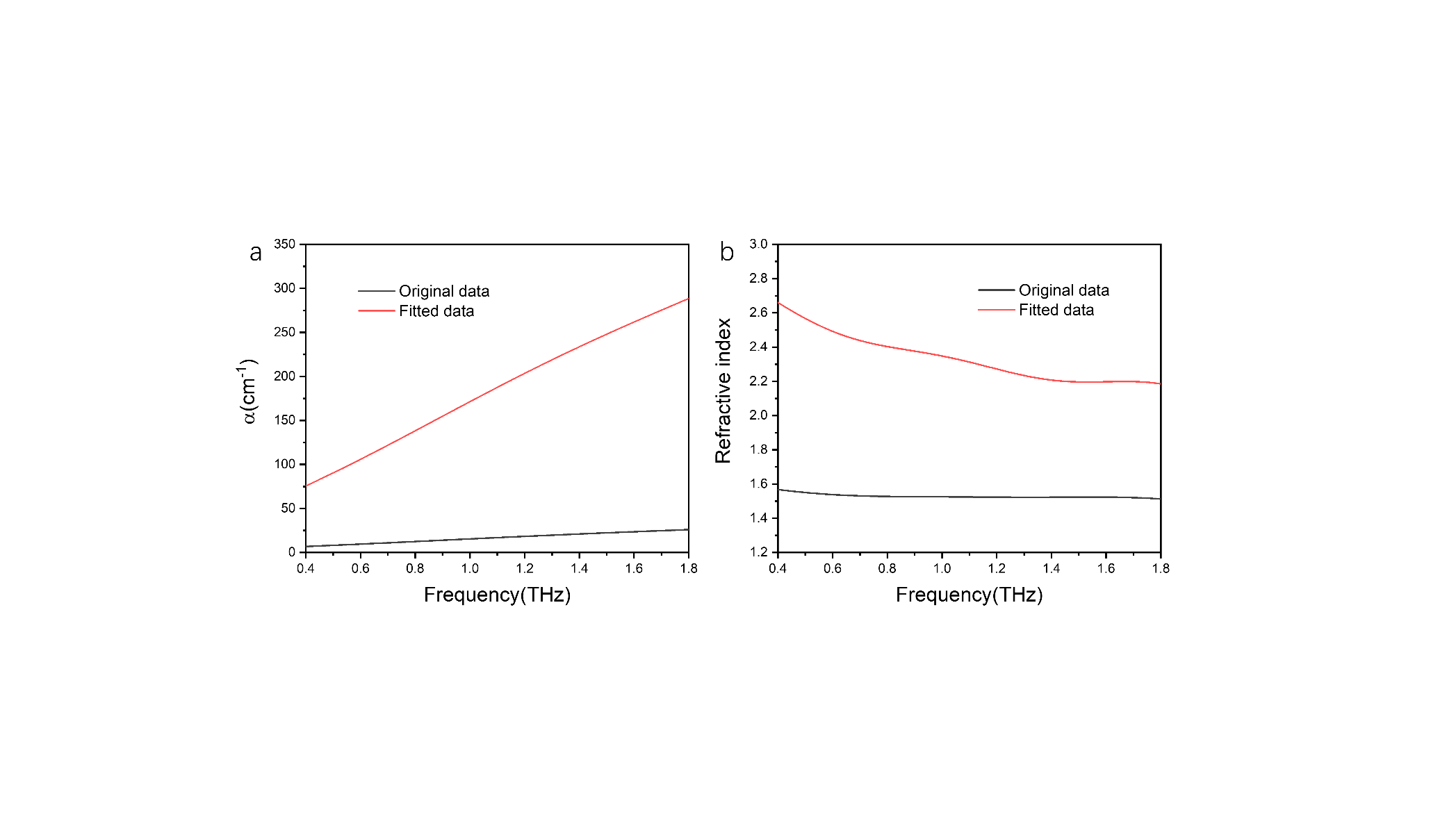


Fig S4. A comparison of the original spectrum and the fitted spectrum of (a) absorption coefficient and (b) refractive index.


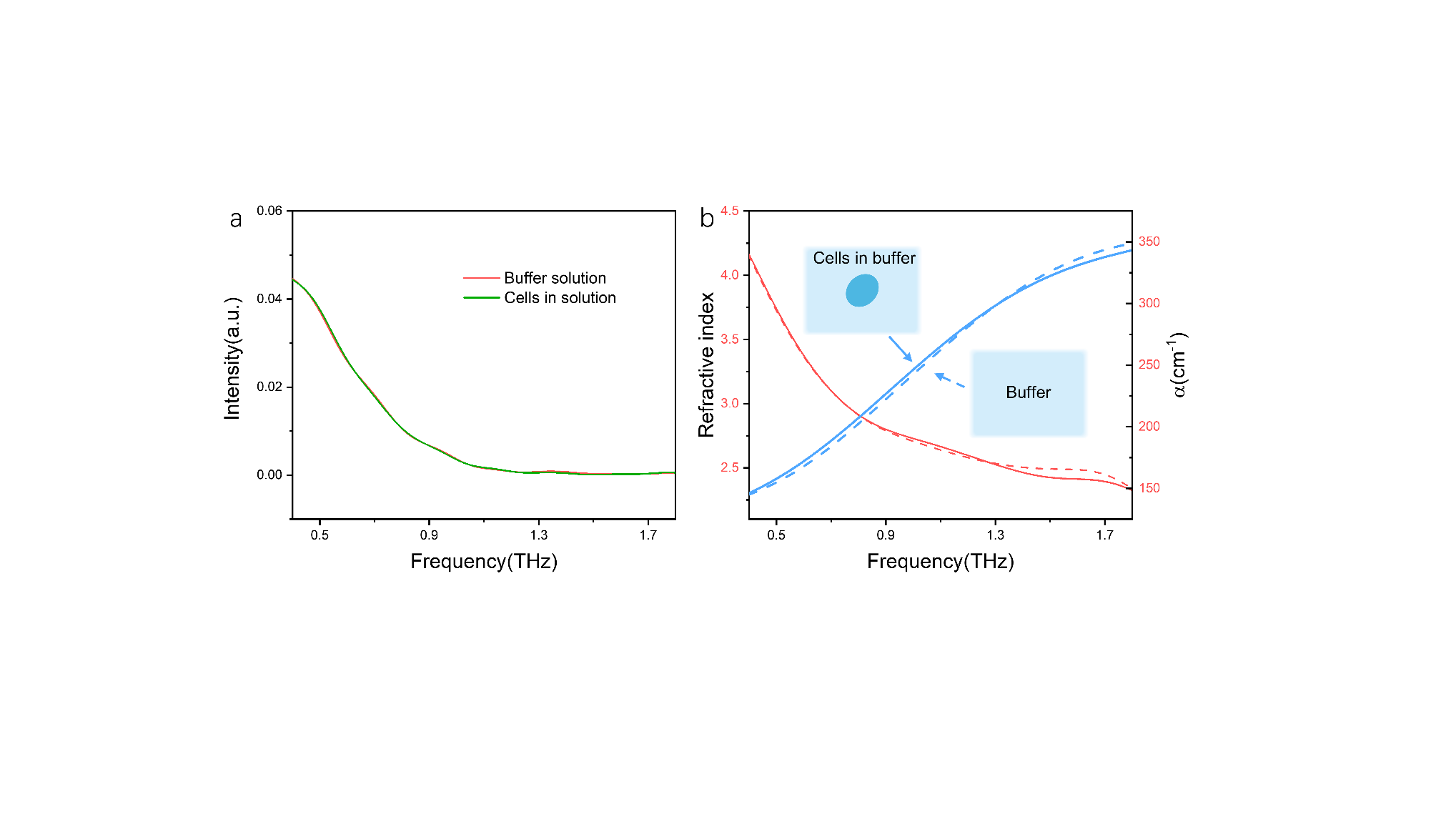


Fig S5. (a) The transmission spectra of aqueous buffer solution and *E. coli* in aqueous buffer solution (cell in solution). (b) The refractive index (left) and absorption spectra (right) of *E. coli*-containing buffer (solid line) and buffer alone (dashed line). The bacterial concentration is 9.0×10^10^ cfu/ml.


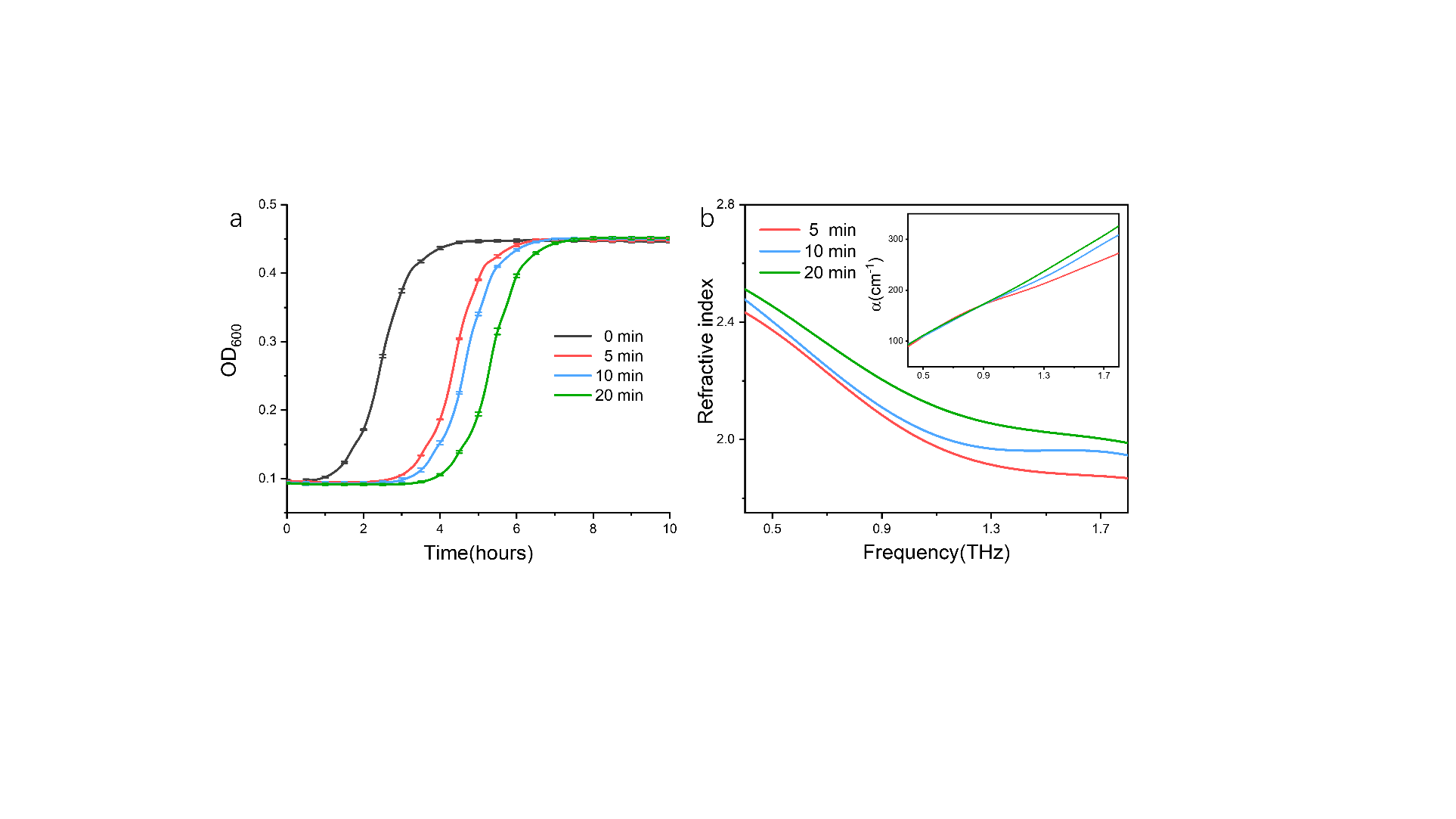


Fig. S6 (a) The growth curve represented by the time course of the optical density at 600 nm wavelength (OD_600_) of *E. coli* treated with 0.4 mM Cu^2+^ for 0, 5, 10 and 20 min; (b) Refractive index spectra of *E. coli* treated with 0.4 mM Cu^2+^ for 5, 10 and 20 min; inset: the corresponding absorption coefficient spectra. All bacteria samples were treated for 20 minutes and set to the same concentration of 9.0×10^10^ cfu/ml before THz measurement.

**
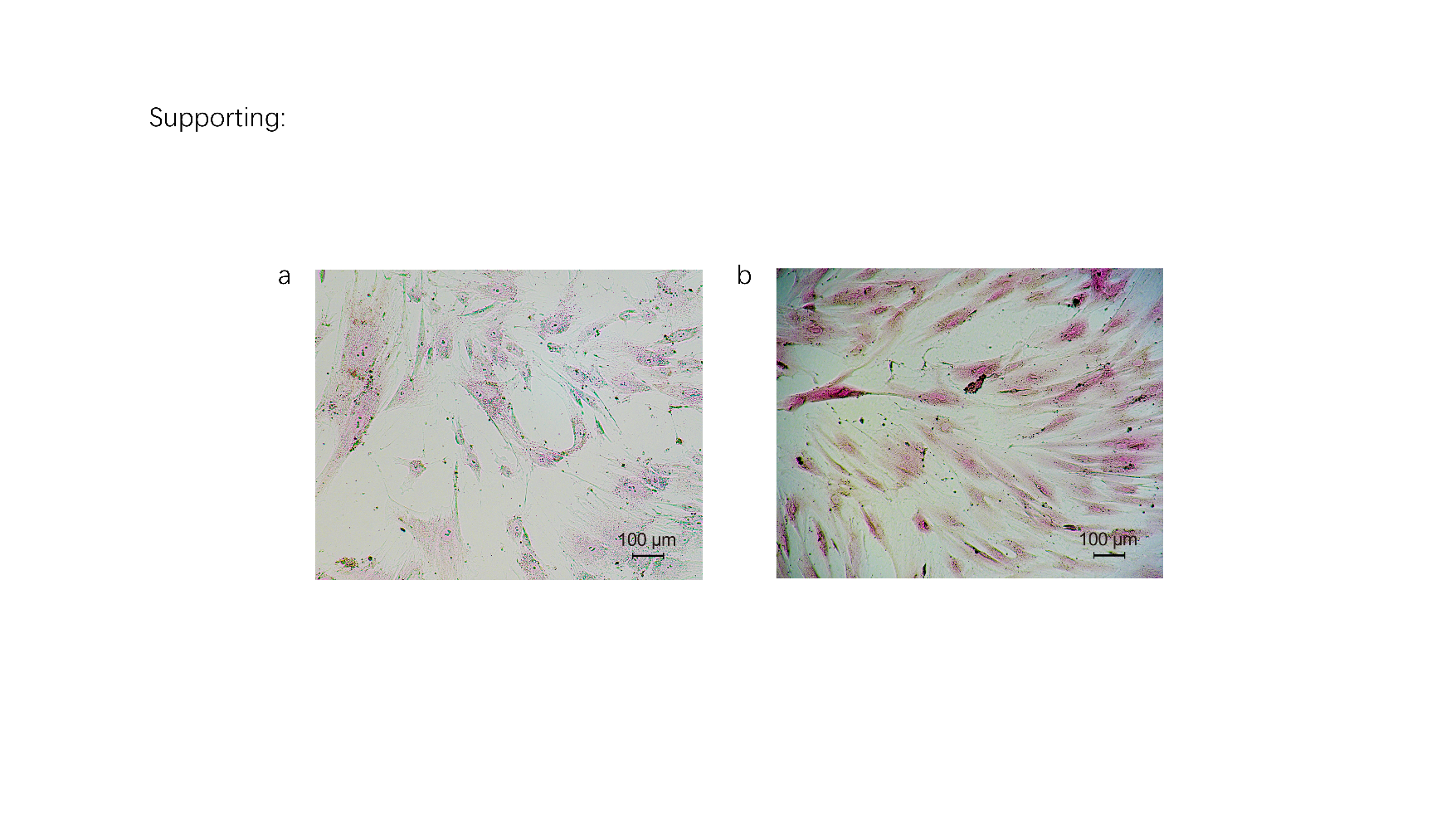
**

Fig. S7. The microscopy photograph of the undifferentiated (a) and osteogenic differentiated (b) MSCs after alizarin red S staining.

**Reference**

1. Scheller, M.; Jansen, C.; Koch, M. Applications of Effective Medium Theories in the Terahertz Regime. In; 2010.

2. Zang, Z.; Li, Z.; Lu, X.; Liang, J.; Wang, J.; Cui, H.-L.; Yan, S., Terahertz spectroscopy for quantification of free water and bound water in leaf. *Computers and Electronics in Agriculture* **2021**, 191, 106515.

3. Hernandez-Cardoso, G. G.; Singh, A. K.; Castro-Camus, E., Empirical comparison between effective medium theory models for the dielectric response of biological tissue at terahertz frequencies. *Appl. Opt.* **2020**, 59, D6-D11.
